# Development and validation of the potential biomarkers based on m6A-related lncRNAs for the predictions of overall survival in the lung adenocarcinoma and differential analysis with cuproptosis

**DOI:** 10.1186/s12859-022-04869-7

**Published:** 2022-08-08

**Authors:** Chen Gao, Ning Kong, Fan Zhang, Liuzhi Zhou, Maosheng Xu, Linyu Wu

**Affiliations:** 1 Department of Radiology, The First Affiliated Hospital of Zhejiang Chinese Medical University (Zhejiang Provincial Hospital of Traditional Chinese Medicine), 54 Youdian Road, Hangzhou, China; 2 The First School of Clinical Medicine, Zhejiang Chinese Medical University, Hangzhou, China; 3 Department of Surgery, The Second Affiliated Hospital, Zhejiang University School of Medicine, Hangzhou, China

**Keywords:** Lung adenocarcinoma, N6-methyladenosine, Long non-coding RNA, Prognosis, Biomarker

## Abstract

**Background:**

The treatment and prognosis of lung adenocarcinoma (LUAD) remains a challenge. The study aimed to conduct a systematic analysis of the predictive capacity of N6-methyladenosine (m6A)-related long non-coding RNAs (lncRNAs) in the prognosis of LUAD.

**Methods:**

594 samples were totally selected from a dataset from The Cancer Genome Atlas. The identification of prognostic m6A-related lncRNAs were performed by Pearson correlation analysis and Cox regression analysis. Systematic analyses, including cluster analysis, survival analysis, and immuno-correlated analysis, were conducted. A prognosis model was built from the optimized subset of m6A-related lncRNAs. The assessment of model was performed by survival analysis, and receiver operating characteristic (ROC) curve. Finally, the risk score of patients with LUAD calculated by the prognosis model was implemented by the analysis of Cox regression. Differential analysis was for further evaluation of the cuproptosis-related genes in two risk sets.

**Results:**

These patients were grouped into two clusters according to the expression levels of 22 prognostic m6A-related lncRNAs. The patients with LUAD in cluster 2 was significantly worse in the overall survival (OS) (*P* = 0.006). Three scores calculated by the ESTIMATE methods in cluster 2 were significantly lower. After the least absolute shrinkage and selection operator algorithm, 10 prognostic m6A-related lncRNAs were totally selected to construct the final model to obtain the risk score. Then the area under the ROC curve of the prognosis model for 1, 3, and 5-year OS was 0.767, 0.709, and 0.736 in the training set, and 0.707, 0.691, and 0.675 in the test set. The OS of the low-risk cohort was significantly higher than that of the high-risk cohort in both the training set (*P* < 0.001) and test set (*P* < 0.001). After the analysis of Cox regression, the risk score [Hazard ratio (HR) = 5.792; *P* < 0.001] and stage (HR = 1.576; *P* < 0.001) were both considered as independent indicators of prognosis for LUAD. The expression levels of five cuproptosis-related genes were significantly different in two risk sets.

**Conclusions:**

The study constructed a predictive model for the OS of patients with LUAD and these OS-related m6A-lncRNAs might have potential roles in LUAD progression.

**Supplementary Information:**

The online version contains supplementary material available at 10.1186/s12859-022-04869-7.

## Background

Lung cancer, a malignancy which has been identified to have the highest mortality and the second highest morbidity worldwidely, has a poor 5-year survival since the rate reaches only 10–20% [1, 2]. The most common subtype is lung adenocarcinoma (LUAD) [3, 4]. Despite therapeutic options including targeted therapy, radiotherapy, chemotherapy and surgery progressed rapidly, the prognosis of LUAD patients remains unsatisfactory [3–5]. LUAD, the pathogenesis of which involves multiple molecular mechanisms through various pathways, requires more in-depth mechanistic research to develop more promising therapies.

N6-methyladenosine (m6A) modification is a crucial RNA post-transcriptional modification in most eukaryotic long non-coding RNAs (lncRNAs) and mRNAs [6, 7]. It can modulate the stabilization, splicing, degradation, translation, and processing of the target RNA via three type of regulators, which include writers (methyltransferases), erasers (demethylases), and readers (binding proteins) [8, 9]. Some studies have indicated that m6A modifications are correlated with the oncogenesis and development of malignancies, including LUAD [10–12]. Wang previously showed that the 13 m6A regulators, such as *KIAA1429* and *FTO*, were aberrantly overexpressed in tumor samples [11]. Moreover, several studies also found that the modification of m6A has involvement in the regulation of the immune response and tumor microenvironment infiltrating cells, such as dendritic cells [13, 14]. Therefore, it is necessary to study these m6A modifications to gain a comprehensive understanding their functions.

LncRNAs, a group of RNAs which is non-coded and has over 200 nucleotides in length, functions in various biological processes including growth, apoptosis, and regulation of cell development [15]. Aberrant regulation of lncRNAs has been investigated and found to be related to different malignancies, including in LUAD [16, 17]. Ding et al. found that high expression of lncRNA *OGFRP1* leads to significantly worse survival outcomes and constructed a prediction model whose area under the receiver operating characteristic (ROC) curve (AUC) reaches 0.766 [18]. Studies focusing on the mechanism by which the m6A modification acts on the occurrence and progression of lncRNA-dependent LUAD are limited and the whole roles of the three types of m6A regulators in the aberrant lncRNAs regulation is still undefined [19]. Separately, the cell death was also involved in the modification of m6A for lncRNAs in the development of LUAD and cuproptosis was new form of cell death, which was found to be associated with the progression of cancer [20–23]. However, the relationship between cuproptosis and m6A-related lncRNAs in the development of LUAD has not been reported.

Therefore, the objective of our research was to perform a bioinformatic analysis to distinguish those survival-related m6A-lncRNAs based on data of LUAD patients from The Cancer Genome Atlas (TCGA) and conduct a systematic analysis. Furthermore, a model was constructed to predict the overall survival (OS) of these patients by utilizing the optimized set of survival-related m6A-lncRNAs. Differential analysis was for further evaluation of the relationship between the performance of the model and cuproptosis.

## Methods

### Datasets and m6A-related genes

The transcriptome profiling normalization data by fragments per kilobase of transcript per million mapped reads and the relevant clinical features were acquired from the Genomic Data Commons Data Portal (https://portal.gdc.cancer.gov). A total of 515 cases with 594 samples were involved in this study from a TCGA project, and 59 samples were in the normal group and 535 in the tumor group. In addition, 23 m6A regulators were totally determined on the basis of previous studies, as shown the Additional file 1, which could be divided in to writers, erasers, and readers. Annotation of the lncRNAs and mRNAs in the TCGA project were conducted using the annotation file of Genome Reference Consortium Human Build 38 (GRCh38) acquired from the GENCODE website (https://www.gencodegenes.org). A total of 14,086 lncRNAs were identified by the Ensemble IDs of the genes in the TCGA dataset.

### Selection of m6A-related lncRNAs and cluster analysis

m6A-related lncRNAs were initially identified by Pearson correlation analysis with the *P* < 0.001 and |Pearson R|> 0.5. Then, the prognostic m6A-related lncRNAs were determined by the univariate analysis of Cox regression. Wilcoxon tests were used for analyzing the prognostic m6A-related lncRNA expression between the normal group and the tumor group. Cluster analysis was used for analyzing the expression of the prognostic m6A-related lncRNAs. The OS of different clusters were compared by Kaplan–Meier (KM) curves and the log-rank test. Then, the prognostic m6A-related lncRNAs expression and clinical features in different clusters were analyzed by differential analysis.

### Differential analysis and correlation analysis of CD274 expression

The CD274 (programmed cell death 1 ligand 1, PD-L1) expression in the normal group and the tumor group, and in the two clusters, were compared using the Wilcoxon test. Moreover, CD274 expression and the expression of prognostic m6A-related lncRNAs were analyzed by correlation analysis.

### Tumor-infiltrating immune cells (TIIC) evaluation and gene set enrichment analysis (GSEA)

According to the CIBERSORT algorithm, standard gene expression data and the Wilcoxon test, the relative proportions and the difference of 22 TIIC subpopulations were evaluated [24]. The immune score, estimate score, and stromal score were computed using the ESTIMATE algorithm to further predict tumor purity and to analyze the tumor microenvironment [25]. The Wilcoxon test was used to analyze these three indicators of the tumor microenvironment between the two clusters. Then we used GSEA software (version 4.1.0) to investigate gene set enrichment in the two clusters of lung adenocarcinoma.

### Establishment of the prognosis model and survival analysis

The patients with LUAD were grouped into a training cohort and a test cohort at a ratio of 5:5 randomly. The optimized subset of prognostic m6A-related lncRNAs was chosen using the least absolute shrinkage and selection operator (LASSO) algorithm to build the final prognosis model. After the number of lncRNAs was determined, the corresponding coefficients of each lncRNA of the optimized subset were calculated. The assessment of prognosis model was then performed by ROC analysis and a risk plot. The natural logarithm of risk score was calculated by summing the chosen lncRNAs expression, weighted by their corresponding coefficients. Then patients with LUAD were divided into low-risk set and high-risk set by the median value of the risk scores. The OS of patients with LUAD in the two risk sets were compared by the KM method and log-rank test in the training cohort and test cohort. The evaluation of the risk score and clinical features for the OS of patients with LUAD were implemented by univariate and multivariate analysis of Cox regression. Then, subgroup analysis of each clinical feature, based on the two risk sets, was conducted using the KM method and log-rank test.

### Differential analysis of risk sets and risk score

The differential analysis of immune score, clinical features and cluster status between the two risk sets were analyzed by chi-squared test. The differential analysis of PD-L1 expression and the cuproptosis-related genes expression were conducted using the Wilcoxon test between the two risk sets. Correlation analysis was also implemented between the risk score and each TIIC of samples using Spearman correlation analysis.

### Statistical analysis

The R statistical software (version 4.0.5) was used for all statistical analyses. The “limma” package was used to assess the transcriptome profiling data. The survival analysis was conducted by the “survival” and, “survminer” packages. The “pheatmap”, “reshape2”, “ggpubr”, and “ggplot2” packages were used to generate heatmaps, boxmaps and risk plots. The “ConsensusClusterPlus” package was to conduct the cluster analysis. The “corrplot” package was to conduct the correlation analysis of PD-L1 expression. The “e1071”, “parallel”, and “preprocessCore” packages were used to evaluate the TIICs. The “estimate” package was to obtain the stromal score, immune score, and estimate score of sample. The “vioplot”, “ggpubr” “ggplot2”, and “ggExtra” packages were used to investigate the differences among the TIICs in the two clusters and to conduct correlation analysis between the risk score and the TIICs. The “caret”, “glmnet”, “timeROC”, “survival”, and “survminer” packages were to build the model and to get the ROC. A two-side *P* < 0.05 was considered statistically significant.

## Results

### Determination of m6A-related lncRNAs in patients with LUAD

**Fig. 1:**
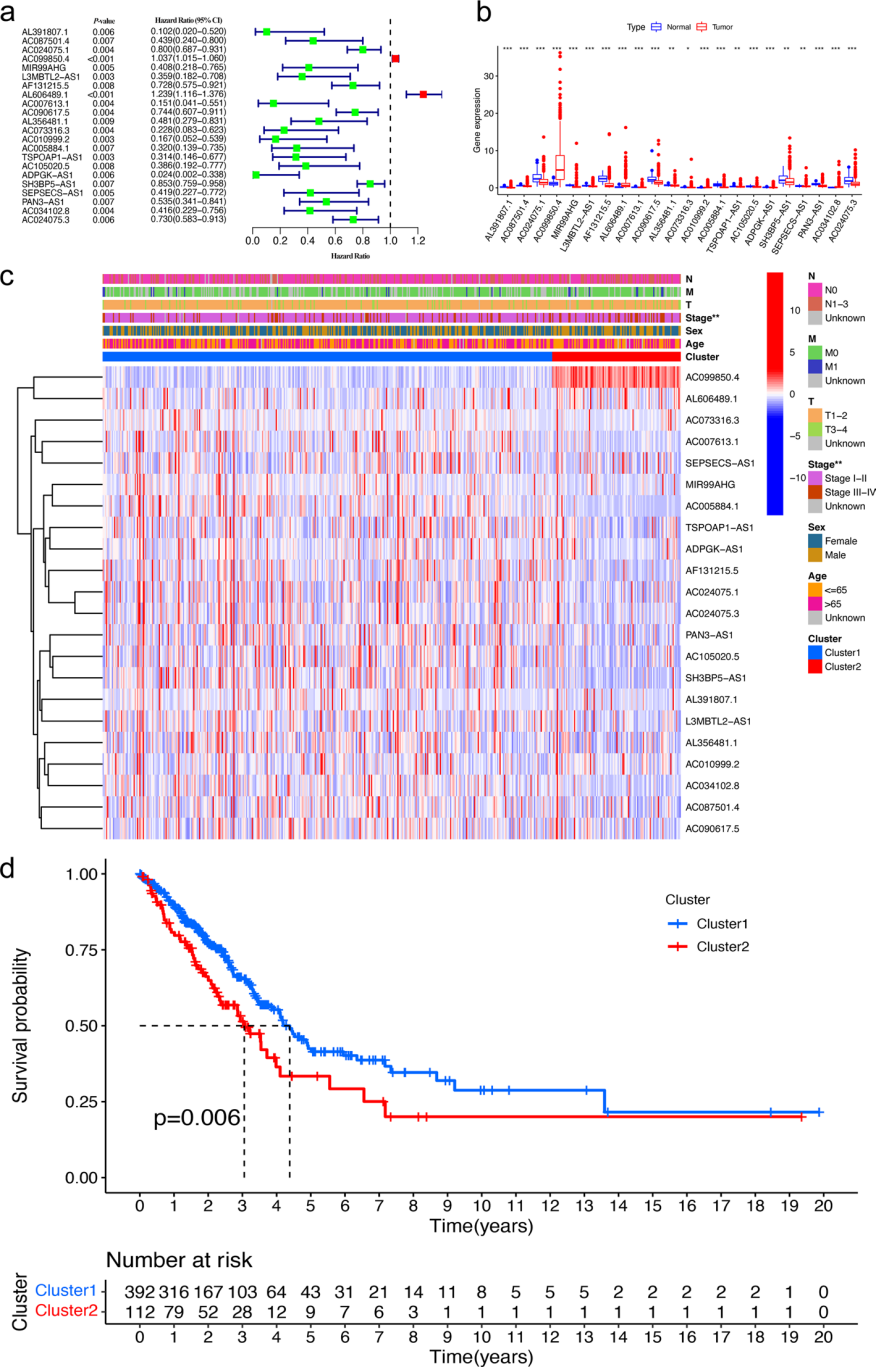
The selection of m6A-related lncRNAs for prognosis and cluster analysis. **a** Forest plot of prognostic m6A-related lncRNAs of patients with LUAD using univariate Cox regression analysis (*P* < 0.01, CI: confidence interval); **b** Boxplot showing the expression levels of prognostic m6A-related lncRNAs in the normal and tumor groups. **c** Heatmap showing the relationship between clinical features, prognostic m6A-related lncRNA expression, and the two clusters. **d** Kaplan–Meier curves showing that cluster 2 group had worse overall survival than cluster 1 by log-rank test (*P* = 0.006). lncRNA, long non-coding RNA; LUAD, lung adenocarcinoma. “***”: *P* < 0.001, “**”: *P* < 0.01, “*”: *P* < 0.05

1558 m6A-related lncRNAs were totally determined using Pearson correlation analysis (with a *P* < 0.001 and |Pearson R|> 0.5). After clinical survival data integrated, 504 patients were further involved in the prognostic analysis from a TCGA project. The information for 504 patients was shown in Table 1. Then, a total of 22 prognostic m6A-related lncRNAs were identified by univariate Cox regression analysis (with *P* < 0.01, Fig. 1a). These prognostic m6A-related lncRNAs had significantly different expression levels between normal and tumor samples by the Wilcoxon test (Fig. 1b). In tumor samples, the expression levels of certain prognostic m6A-related lncRNAs, such as *AC099850.4*, *AL606489.1*, *AC010999.2*, and *AC034102.8*, were higher than in normal samples; however, the expression levels of other prognostic m6A-related lncRNAs, such as *PAN3-AS1*, *AF131215.*5, *AC024075.1*, *MIR99AHG*, *AC005884.1*, and *AC090617.5*, were lower than in normal samples (Fig. 1b).

**Table 1:** Clinical features of 504 patients with LUAD

Clinical features	TCGA database (n = 504)
Age	65.30 ± 10.03
*Sex*	
Male	234
Female	270
*Pathological Stage*	
I	270
II	119
III	81
IV	26
Unknown	8
*T*	
T1	168
T2	269
T3	45
T4	19
Unknown	3
*N*	
N0	325
N1	94
N2	71
N3	2
Unknown	12
*M*	
M0	335
M1	25
Unknown	144

### Cluster analysis of m6A-related lncRNAs

According to the expression of the 22 prognostic m6A-related lncRNAs, all samples were divided into two clusters by cluster analysis. Then, the differences in clinical features and the 22 prognostic m6A-related lncRNAs between the two clusters were investigated. A heatmap showed that the stage classification was significantly different between the two clusters (Fig. 1c). Moreover, the OS between two clusters were compared. As shown in Fig. 1d, the OS of patients with LUAD was significantly different between the two clusters (*P* = 0.006).

### Immuno-correlated analysis

**Fig. 2:**
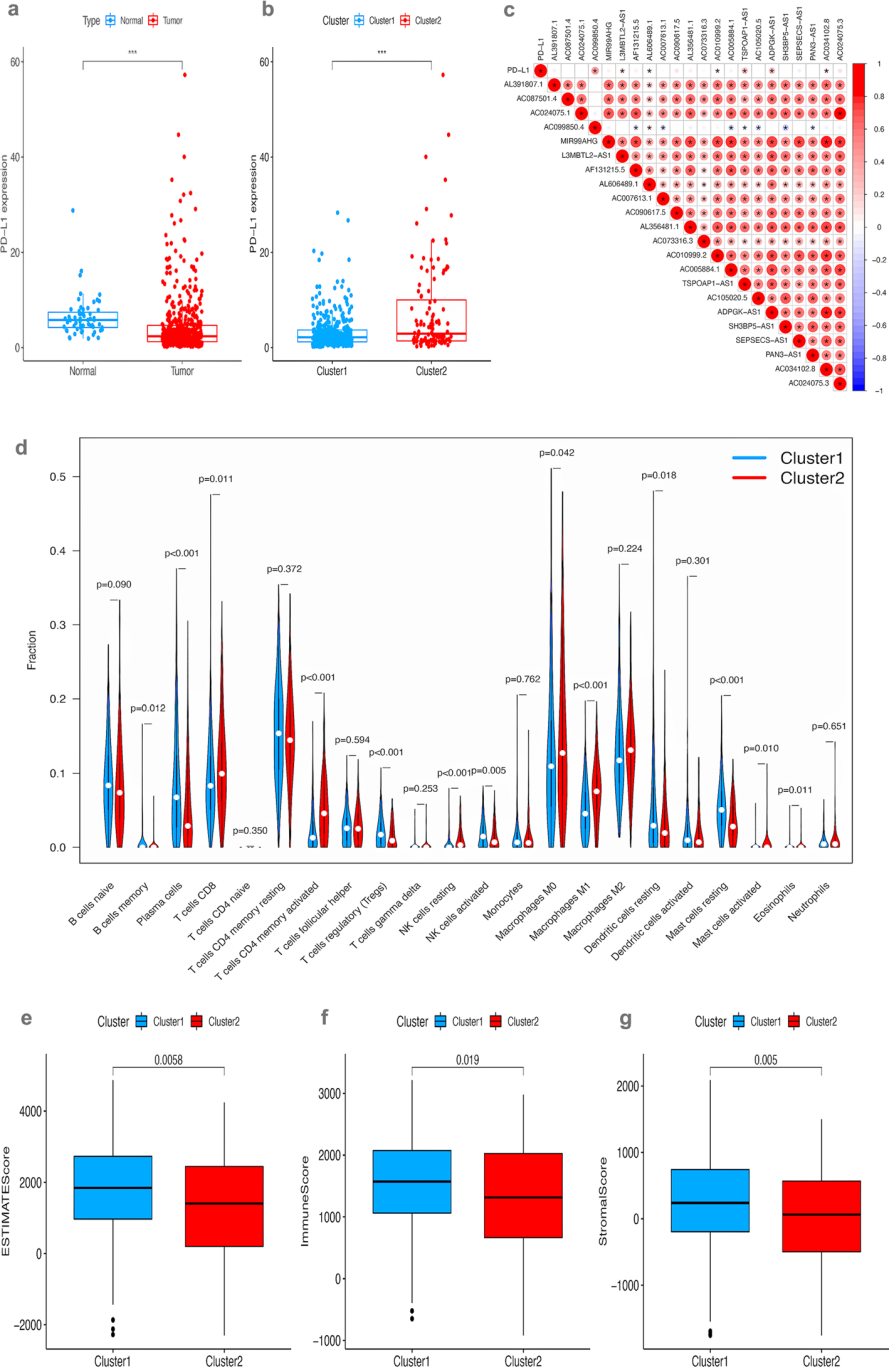
Immuno-correlated analysis. **a** Boxplot showing that the CD274 (PD-L1) expression was significantly different between the normal set and the tumor set. ***: *P* < 0.001. **b** Boxplot showing that PD-L1 expression was significantly different between the two clusters. ***: *P* < 0.001. **c** Plot showing the correlation between CD274 (PD-L1) expression and the expression of 22 prognostic m6A-related lncRNA. The red circle indicates a positive correlation and the blue circle indicates a negative correlation. “”: *P* < 0.05. **d** Violin plot showing the differences in the percentage of 22 kinds of tumor-infiltrating immune cells between the two clusters. **e**, **f**, **g**: These boxplots show that the estimate score, immune score, and stromal score of cluster 2 were significantly lower than cluster 1 (*P* = 0.0058, 0.019, and 0.005, respectively). PD-L1, programmed cell death 1 ligand 1; lncRNA, long non-coding RNA

The PD-L1 expression in the tumor group was significantly lower than that in the normal group (Fig. 2a). The PD-L1 expression of cluster 1 was significantly lower than that in cluster 2 (Fig. 2b). Figure 2c shows the correlation between PD-L1 expression and the expression levels of the 22 prognostic m6A-related lncRNAs. The infiltration of 22 immune cells in the LUAD microenvironment were analyzed based on the CIBERSORT algorithm. As shown in Fig. 2d, the 22 kinds of TIICs were compared between the two clusters. The violin plot showed that the proportions of 13 kinds of immune cells were significantly different between the two clusters. Then the stromal score, immune score, and estimate score assessed by the ESTIMATE methods were compared between two clusters and all three were significantly different (Fig. 2e–g).

### GSEA analysis

**Fig. 3:**
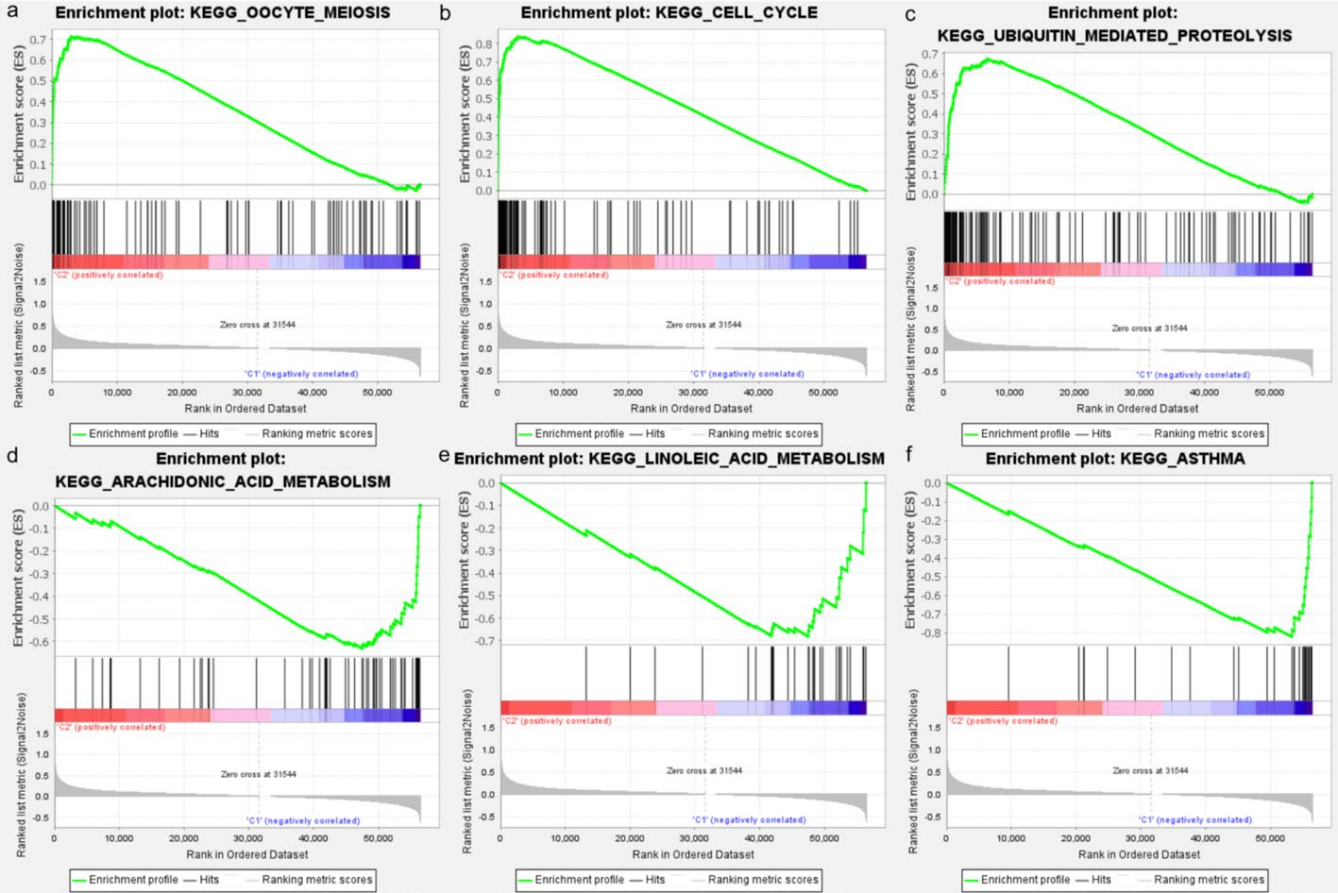
Gene set enrichment analysis of the two clusters. **a**–**c**: The top three gene sets enriched in cluster 2 included oocyte meiosis (NES = 2.65, Norm *P* < 0.001, FDR q < 0.001), cell cycle (NES = 2.60, Norm *P* < 0.001, FDR q < 0.001), and ubiquitin mediated proteolysis (NES = 2.52, Norm *P* < 0.001, FDR q < 0.001). **d**–**f**: The top three gene sets enriched in cluster 1 included arachidonic acid metabolism (NES =  − 2.11, Norm *P* < 0.001, FDR q = 0.014), linoleic acid metabolism (NES =  − 2.08, Norm *P* < 0.001, FDR q = 0.013) and Asthma (NES =  − 2.07, Norm *P* = 0.002, FDR q = 0.009). NES, normalized enrichment score; Norm, normalized; FDR, false discovery rate

GSEA was conducted and the results showed that in cluster 2, the genes set was mainly enriched for the oocyte meiosis, cell cycle, and ubiquitin mediated proteolysis (Fig. 3a–c). In cluster 1, the genes set was mainly enriched for arachidonic acid metabolism, linoleic acid metabolism, and asthma (Fig. 3d–f).

### Construction of the prognosis model and survival analysis

**Fig. 4:**
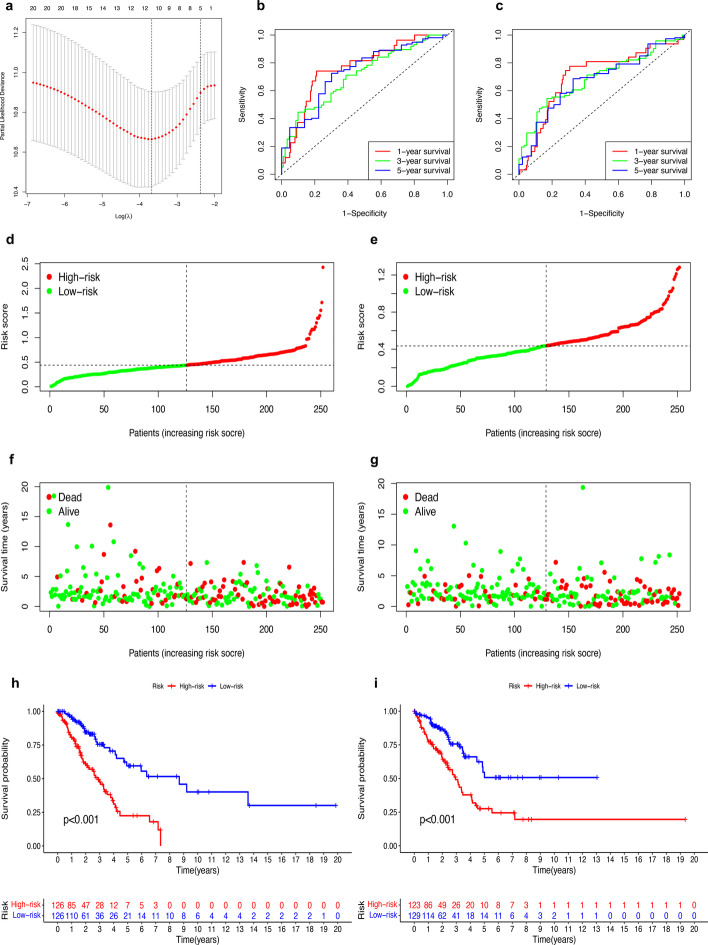
Building the prognosis model and survival analysis. **a** The process of least absolute shrinkage and selection operator regression to establish the final prognosis model. **b**, **c** The receiver operating characteristic of the prognosis model for OS in the training cohort (**b**) and validation cohort (**c**). **d**, **e** Patients were grouped into two risk sets by mean value of the risk score of training cohort in the training cohort (**d**) and validation cohort (**e**). **f**, **g** The distribution of patients with survival status based on two risk groups in the training cohort (**f**) and validation cohort (**g**). **h**, **i**: Kaplan–Meier curves of two risk sets in the training cohort (**h**) and validation cohort (**i**)

The 504 patients were randomly grouped into a training cohort and a validation cohort. Then 10 prognostic m6A-related lncRNAs chosen by the LASSO algorithm were to obtain the risk score, including *AC087501.4*, *L3MBTL2-AS1*, *AL606489.1*, *AC007613.1*, *AC090617.5*, *AC073316.3*, *AC010999.2*, *AC005884.1*, *TSPOAP1-AS1*, and *ADPGK-AS1*(Fig. 4a). The formula is shown in the Additional file 2. Built by the LASSO algorithm, the AUC of the prognosis model for 1-year, 3-year, 5-year OS was 0.767, 0.709, and 0.736 in the training cohort and 0.707, 0.691, and 0.675 in the validation cohort (Fig. 4b–c). Then, according to the median value of the risk scores of the training cohort, the survival status of the training cohort and the validation cohort were analyzed based the two risk sets (Fig. 4d–g). Moreover, the OS of low-risk set were significantly higher than the high-risk set in both the training cohort (*P* < 0.001) and validation cohort (*P* < 0.001) (Fig. 4h–i).

### Cox regression analysis and subgroup analysis

**Fig. 5:**
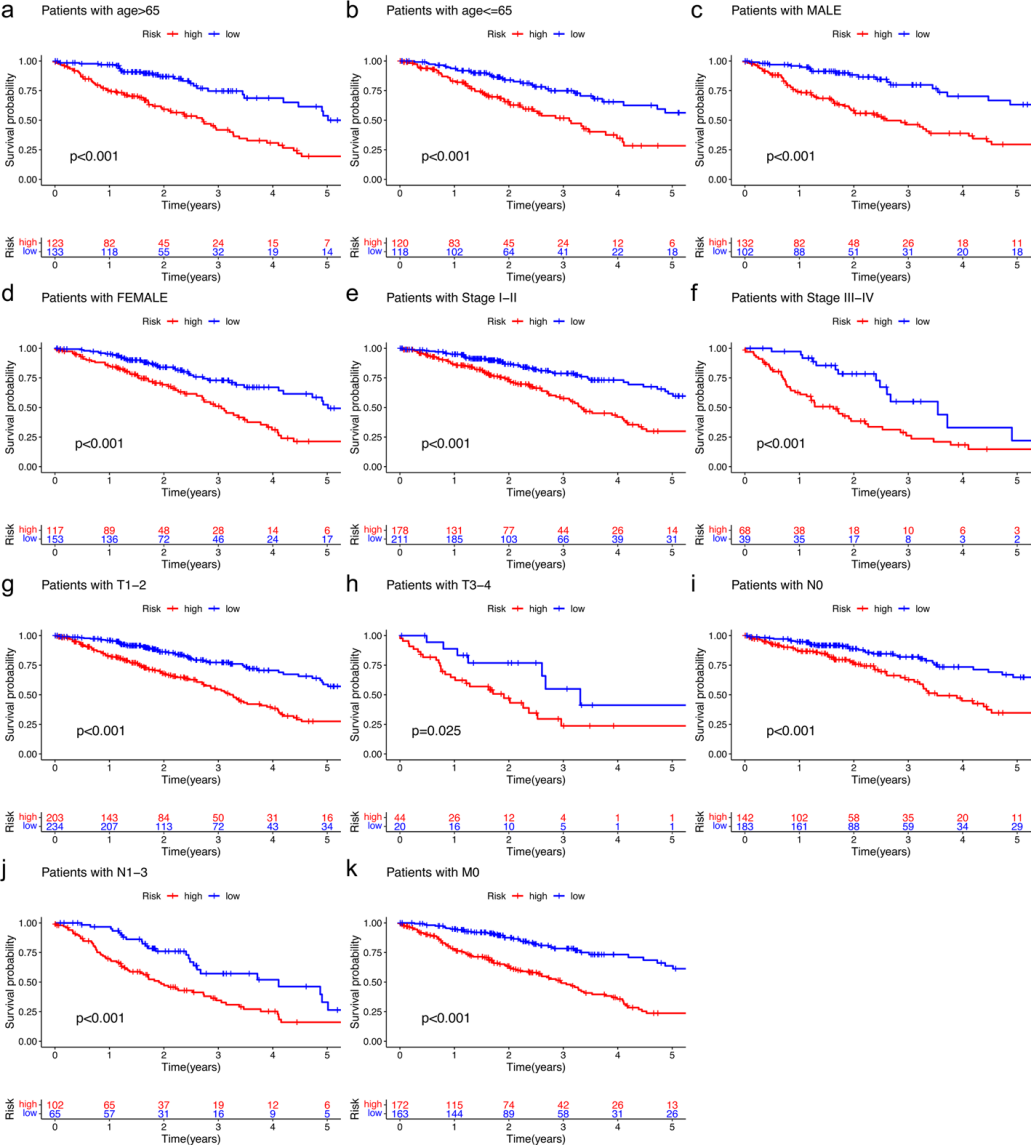
Survival analysis of clinical subgroups of patients. **a**–**k**: Kaplan–Meier curves and the log-rank test showed that the overall survival of the high-risk set was significantly worse than that of the low-risk set in these 11 subgroups of patients (**a**–**k**)

**Fig. 6:**
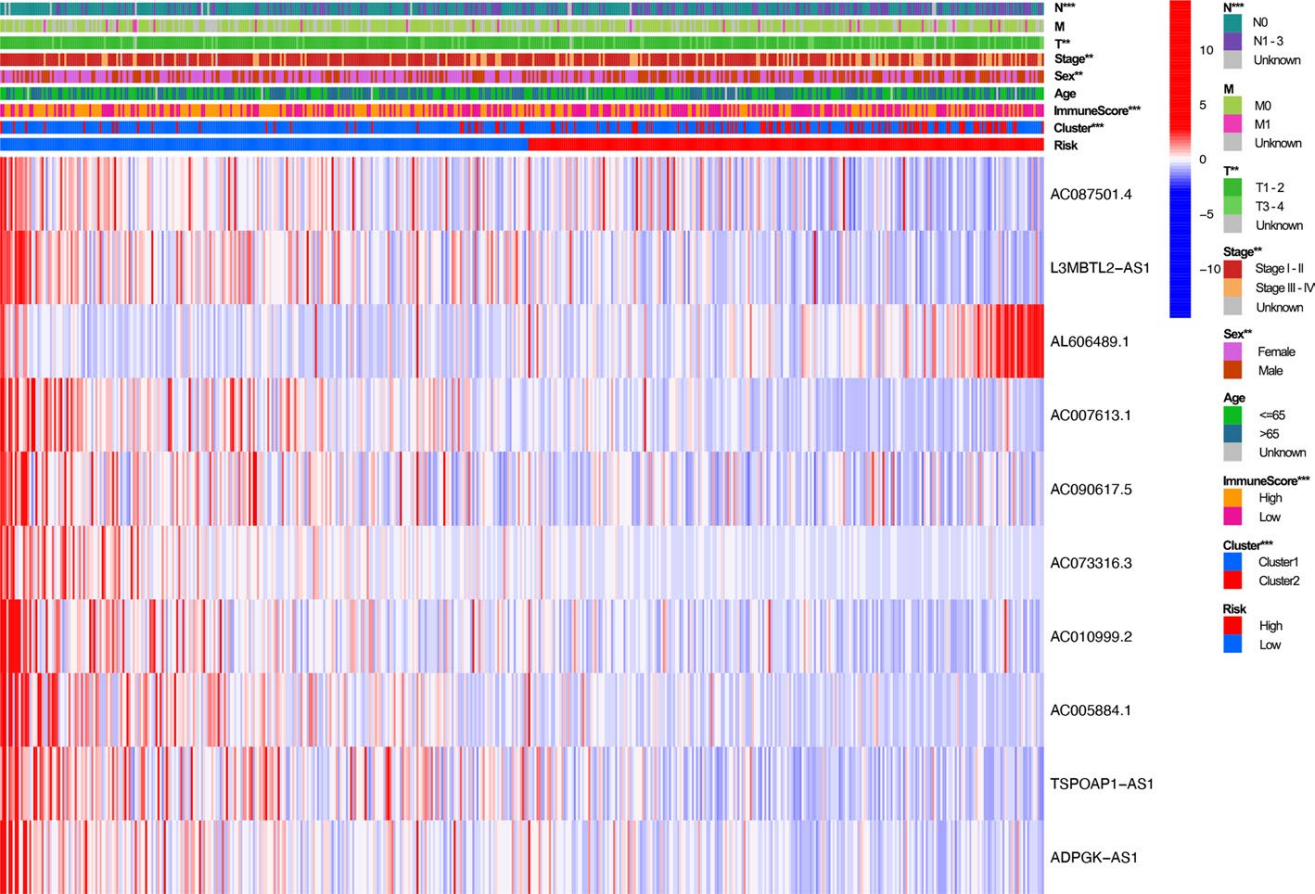
Heatmap analysis of the two risk sets in all patients.The figure shows that the differences of patient with N stage, T stage, Stage, Sex, Immune Score, and Cluster was respectively significant in two risk sets. The heatmap also shows the expression of the 10 prognostic m6A-related lncRNAs in the two risk sets.lncRNA, long non-coding RNA.“***”: *P* < 0.001, “**”: *P* < 0.01, “*”: *P* < 0.05

**Fig. 7:**
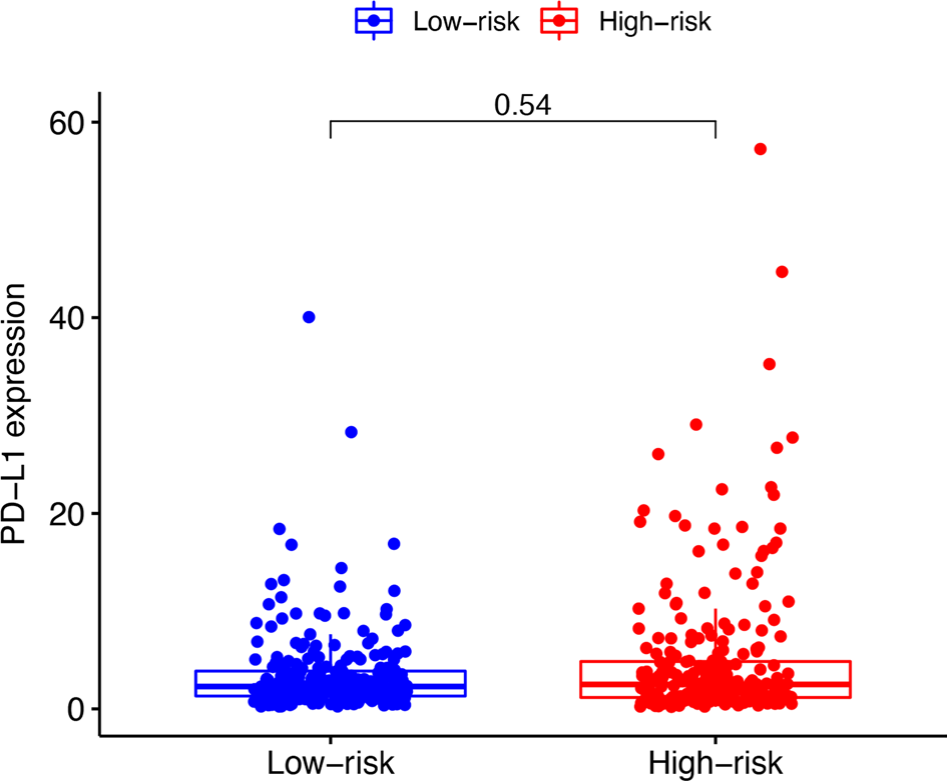
The difference of CD274 (PD-L1) expression in the two risk sets. The boxplot shows that the PD-L1 expression of the high-risk set and low-risk set were no significant difference (*P* = 0.54). PD-L1, programmed cell death 1 ligand 1

**Fig. 8:**
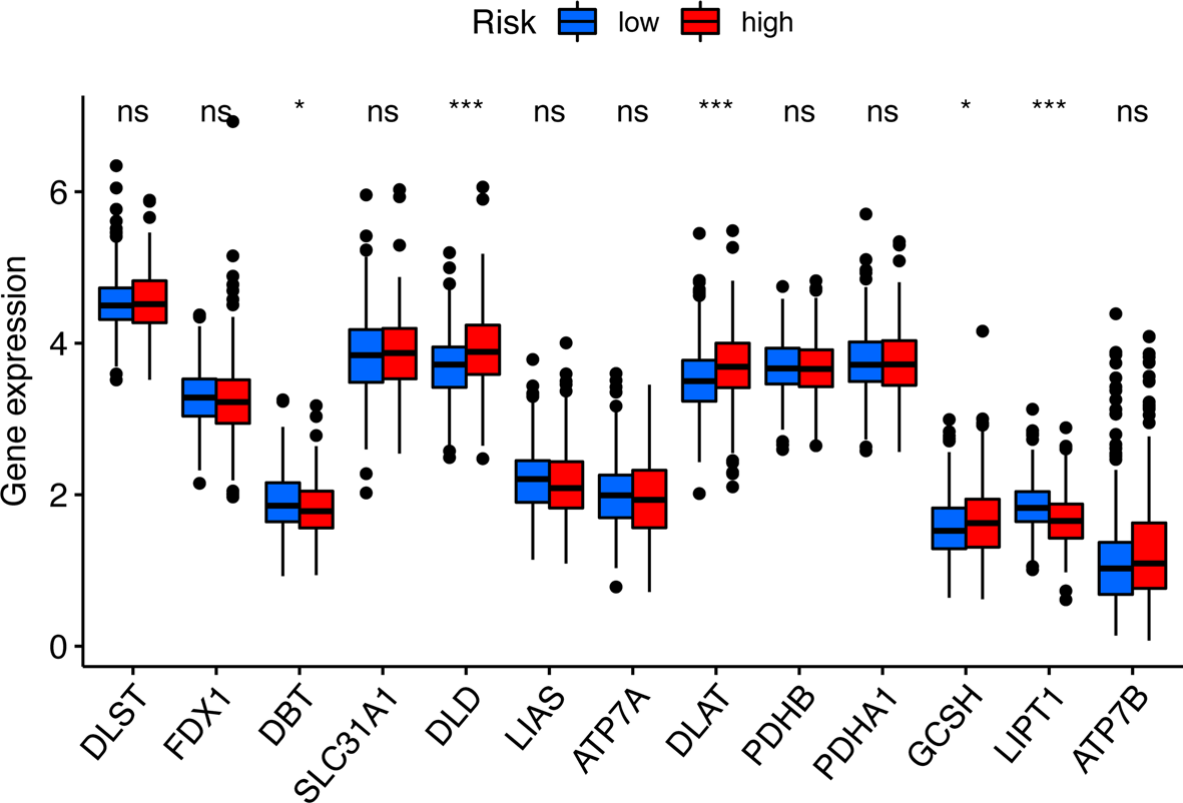
Differential analysis of cuproptosis-related genes in the two risk sets.The boxplot shows that the difference of the expression of five cuproptosis-related genes were significant in the two risk sets. Log-transformation was only performed to draw the boxplot for visualization.“ns”: not significant; “***”: *P* < 0.001; “**”: *P* < 0.01; “*”: *P* < 0.05

**Fig. 9:**
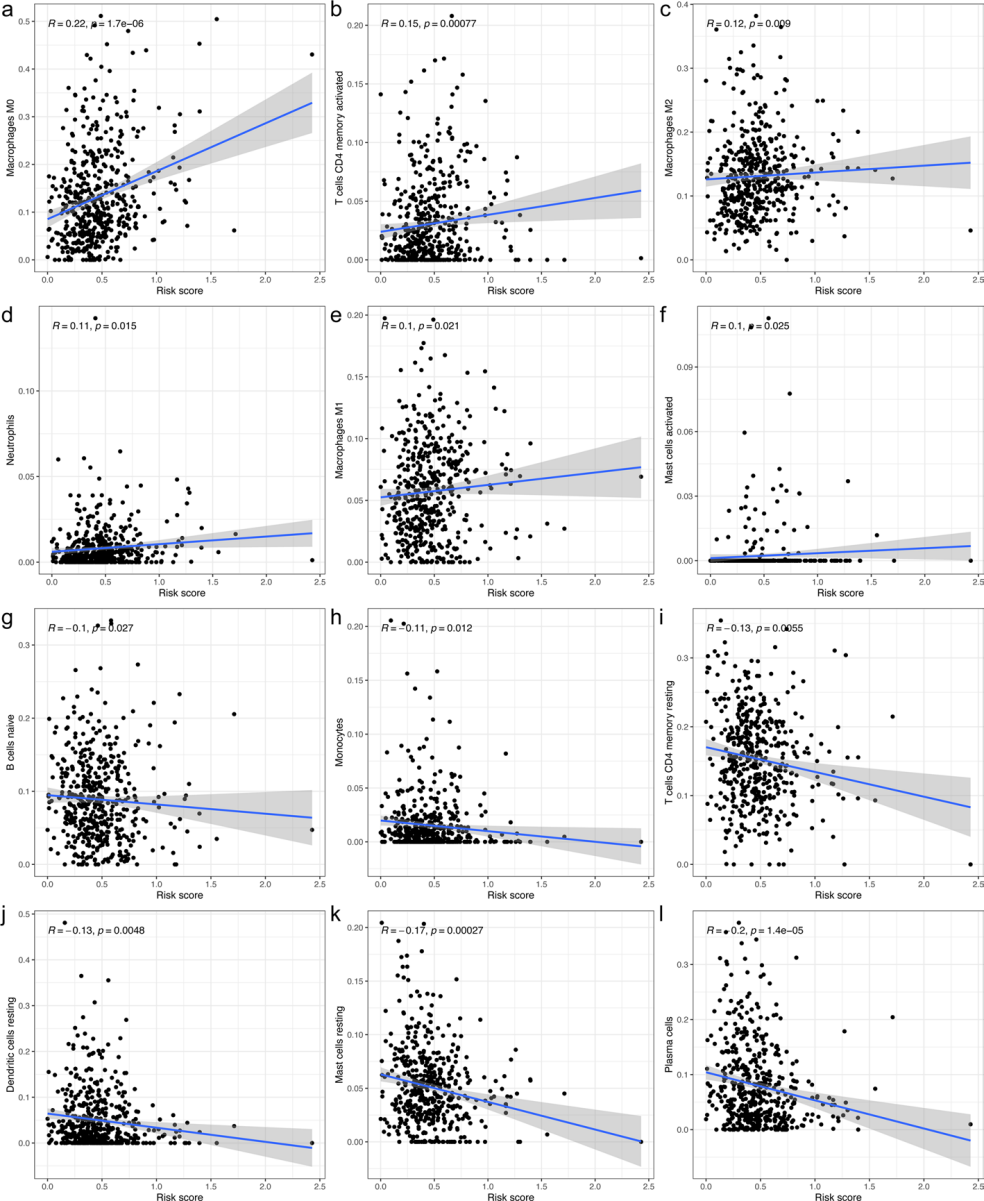
The correlation between immune cells of samples and the risk score.The figure shows 12 types of immune cells from samples with significantly related with risk scores

After univariate and multivariate analysis of Cox regression, the risk score and stage were both considered as independent predictive factors for the OS of patients with LUAD and the risk score was the key one in all groups (Table 2). After the subgroup analysis, the low-risk set showed significantly higher OS than the high-risk set in these 11 subgroup patients, as shown in Fig. 5. Then, T stage, TNM stage, N stage, sex, immune score, and cluster of patients were significantly different in the two risk groups (Fig. 6). Figure 6 also showed the difference expression levels of the 10 prognostic m6A-related lncRNAs in the two risk groups. The PD-L1 expression levels of the low-risk set and high-risk set were not significantly different (*P* = 0.54), as shown in the Fig. 7. The expression levels of five cuproptosis-related genes were significantly different in two risk sets, as shown in the Fig. 8. Moreover, the 6 kinds of immune cells, such as plasma cells (R =  − 0.2), correlated significantly and negatively with the risk score, whereas the other 6 kinds of immune cells, such as M0 macrophages (R = 0.22), correlated significantly and positively with the risk score (Fig. 9).

**Table 2:** Univariate and multivariate Cox regression analysis

Variable	Univariate Cox regression		Multivariate Cox regression	
	HR (95% CI)	*P* value	HR (95% CI)	*P* value
*Training cohort*				
Age	1.012 (0.989–1.035)	0.310	1.011 (0.988–1.034)	0.342
Sex	1.185 (0.785–1.790)	0.419	0.820 (0.529–1.271)	0.374
Stage	1.629 (1.347–1.969)	< 0.001	1.549 (1.269–1.892)	< 0.001
Risk score	6.338 (3.749–10.716)	< 0.001	5.952 (3.338–10.615)	< 0.001
*Test cohort*				
Age	1.003 (0.982–1.024)	0.781	1.011 (0.990–1.032)	0.299
Sex	1.039 (0.681–1.585)	0.859	1.053 (0.689–1.609)	0.811
Stage	1.639 (1.335–2.013)	< 0.001	1.576 (1.266–1.961)	< 0.001
Risk score	7.555 (3.530–16.170)	< 0.001	5.792 (2.707–12.396)	< 0.001

*CI* confidence interval, *HR* Hazard ratio

## Discussion

The transcriptome information and clinical data of patients that have been diagnosed with LUAD were totally collected from the TCGA dataset, with the aim to investigate those m6A-related lncRNAs that associated with LUAD prognosis. After the analysis of univariate Cox regression, 22 prognostic m6A-related lncRNAs were confirmed and used for clusters analysis. The OS of the patients in cluster 1 was significantly higher than in cluster 2. Then 10 m6A-related lncRNAs were selected from the previously mentioned 22 prognostic biomarkers, based on which a prediction model aiming at the OS of patients was established. The low-risk set patients with LUAD had significantly higher OS than the other set (*P* < 0.001). The risk score was considered as an independent risk factor for OS after the univariate and multivariate analyses of Cox regression. In the later subgroup analysis, the low-risk set had a significantly higher OS revealed by groups with M0 stage and divided by sex, age, TNM stage, N stage, and T stage. Moreover, the difference of T stages, TNM stages, N stages, sex, immune scores, five cuproptosis-related genes and clusters of these patients were significant in two risk sets.

Researches have shown that m6A modification of lncRNAs could regulate the oncogenesis and progression of malignancies [26–28]. Lan et al. showed that *VIRMA*, which was also named *KIAA1429*, could regulate lncRNA *GATA3* to drive hepatocarcinogenesis, progression, and metastasis based on an m6A modification [26]. Yang et al. showed that *METTL14*, which is one of m6A “writer” regulators, could increase the m6A-methylated rate of lncRNA *XIST* and decrease the levels of *XIST* to suppress the proliferation and metastasis of colorectal cancer cells [27]. Ni et al. showed that *YTHDF3* could promote m6A-related lncRNA *GA55* degradation and then accelerate the development of colorectal cancer [28]. These studies indicated that m6A-related lncRNAs could contribute to not only the oncogenesis but also progression of malignant tumors and lncRNAs might be involved in competing against endogenous RNAs, affecting the invasiveness of tumors. However, taking a thorough look at how m6A modifications act on the progression of lncRNA-dependent LUAD is challengeable. Our present study found the gene set of m6A-related lncRNAs in LUAD mainly enriched in the oocyte meiosis, cell cycle, and ubiquitin mediated proteolysis in cluster 2, whose OS was obviously poorer than in cluster 1. These results indicated that lncRNAs with the m6A modification could influence the progression of LUAD and then the OS of patients with LUAD. Therefore, we believe these lncRNAs will be confirmed as potential therapeutic targets of LUAD by studying the functions and interaction of these lncRNAs and their m6A modifications in a future study.

In the present study, 10 of 22 m6A-related lncRNAs were selected by the LASSO algorithm, including *AC087501.4*, *L3MBTL2-AS1*, *AL606489.1*, *AC007613.1*, *AC090617.5*, *AC073316.3*, *AC010999.2*, *AC005884.1*, *TSPOAP1-AS*1, and *ADPGK-AS1*, which could predict the prognosis of LUAD. Several of the 10 lncRNAs have been investigated in different tumors [29–31]. Giulietti et al. showed that the methylation level of the *TSPOAP1-AS1* promoter was higher in pancreatic ductal adenocarcinoma than in normal tissues and the aberrant methylation level of the lncRNA might be considered as an indicator for the diagnosis of pancreatic ductal adenocarcinoma [29]. Yang et al. showed that the expression level *ADPGK-AS1* in the adjacent non-tumor tissues of patients with breast cancer was lower than in the tumor tissues, which might be regarded as an indicator for the prognosis of breast cancer patients because *ADPGK-AS1* could facilitate cell proliferation and migration, suppress cell apoptosis, and induce epithelial-mesenchymal transition [30]. The study of Xu et al. showed that the risk score comprised of 12 m6A-related lncRNAs could predict the 1-year OS of patients with LUAD, and the AUC of its risk score was 0.759 [19]. The group of 12 m6A-related lncRNAs was different with our study and there might be some reasons as below. Firstly, our study included 23 m6A-related genes but the study of Xu et al. only included 21 m6A-related genes [19]. Therefore, our study performed further analysis with two more m6A-related genes than the study of Xu et al. [19]. Secondly, the Pearson's correlation coefficient of their study and our study was more than 0.3 and 0.5, respectively [19]. Thirdly, the prognosis of m6A-related lncRNAs selected by univariate Cox regression analysis in their study and our study was different (*P* < 0.05 and *P* < 0.01, respectively) [19]. Fourthly, sample size was insufficient. Moreover, the version of the annotation files might be different. What’s more, the study of Zhao et al. used 13 m6A-related lncRNAs to construct the prediction model of patients with LUAD [32]. The two studies were similar to some extent, but there were some differences between the results of our study and the study of Zhao et al. [32]. The main reasons are as follows. Firstly, 3 of 23 m6A-related genes are different in our study and the study of Zhao et al. [32]. IGF2BP1, IGF2BP2, IGF2BP3 were also included in the study of Xu et al., which are consistent with our study but not with the study of Zhao et al. [19, 32]. Therefore, there are different in m6A-related genes between our study and the study of Zhao et al. [32]. Secondly, there are different in m6A-related lncRNAs between our study and the study of Zhao et al. because of the above mentioned the differences of m6A-related genes [32]. Thirdly, a total of 504 patients were included into our study to construct the model, but 468 patients were included into the study of Zhao et al. [32]. Although two studies were derived from the TCGA lung adenocarcinoma dataset, there were differences in the transcriptome data due to the inconsistency of the patients included. Fourthly, 22 m6A- related lncRNAs were obtained by the univariate Cox regression analysis (with *P* < 0.01) in our study, while 91 m6A- related lncRNAs were obtained by the univariate Cox regression analysis (with *P* < 0.05) in the study of Zhao et al. [32]. Although there have been few studies about the relationship of how m6A-related lncRNAs act on the occurrence and progression in LUAD, based on our results, we still believe these m6A-lncRNAs have roles in LUAD tumorigenesis and progression, which need to be confirmed in future studies.

Several studies have investigated the relationship between TIICs and the prognosis of lung cancer [33–35]. Li et al. found that in the low-risk set, the percentage of neutrophil infiltration was lower while patients in the high-risk set had poorer prognosis in nonsquamous non-small cell lung cancer [33]. Sun et al. showed that LUAD groups with lower immune score, stromal score, or estimate scores could have worse OS than those with higher scores [34]. The finding is in accordance with the results of this study. We observed no difference in PD-L1 expression between the two risk sets. Similarly, Zhang et al. showed that there was no significant difference in PD-L1 expression among patients with adenocarcinoma in situ, minimally invasive adenocarcinoma, and invasive adenocarcinoma and this finding is also in line with ours [35]. However, evidence suggest that immune checkpoint inhibitors targeting the PD-1/PD-L1 interaction could lead to a reversal of the lung cancer–induced immunosuppressive microenvironment, bringing about an effective host antitumor immune response and also significant improvement in survival [36–38]. Therefore, further research into the undying association between the CD274 (PD-L1) expression and prognosis of LUAD is required. Moreover, our study showed that the expression levels of five cuproptosis-related genes were significantly different in two risk sets. The result indicated cuproptosis might have the involvement in the modification of m6A for lncRNAs in the development of LUAD, which might help to identify tumor therapeutic targets, and the further investigation is also required.

Several limitations are worth mentioning. Firstly, the study only included a TCGA dataset and the prognostic m6A-related lncRNAs need to be validated using various datasets or independent LUAD cohorts in further research. Secondly, the detailed mechanism and function of the prognostic m6A-related lncRNAs in tumorigenesis and the progression of LUAD need to be confirmed by in vitro and in vivo experiments.

## Conclusions

In conclusion, the present study investigated the association between m6A-related lncRNAs and the prognosis in patients with LUAD, establishing a prognosis model for the OS of these patients.

The difference in the expression levels of five cuproptosis-related genes were confirmed in two risk sets. These results suggested potential therapeutic targets of LUAD, which might be confirmed by studying the functions and mechanism of m6A-related lncRNAs in the future.

## Supplementary Information

**Additional file 1**. The official full name of 23 m6A regulators.

**Additional file 2**. The formula of risk score.

**Additional file 3**. The data analysis.

## Data Availability

All data, including the transcriptome profiling normalization data and the relevant clinical features, were acquired from the Genomic Data Commons Data Portal of TCGA (https://portal.gdc.cancer.gov/repository), which is the open access data. The data analysis was also included into the Additional file 3.

## Abbreviations

AUC: Area under the receiver operating characteristic curve
CI: Confidence interval
FDR: False discovery rate
GRCh38: Genome reference consortium human build 38
GSEA: Gene set enrichment analysis
HR: Hazard ratio
LASSO: Least absolute shrinkage and selection operator
lncRNAs: Long non-coding RNAs
LUAD: Lung adenocarcinoma
m6A: N6-methyladenosine
NES: Normalized enrichment score
Norm: Normalized
OS: Overall survival
PD-L1: Programmed cell death 1 ligand 1
ROC: Receiver operating characteristic
TCGA: The Cancer Genome Atlas
TIIC: Tumor-infiltrating immune cells
KM: Kaplan–Meier
