# Supplementary material for: Development and validation of the potential biomarkers based on m6A-related lncRNAs for the predictions of overall survival in the lung adenocarcinoma and differential analysis with cuproptosis
Source: BMC Bioinformatics. 2022 Aug 8;23:327. doi: 10.1186/s12859-022-04869-7 (PMC9358839; doi:10.1186/s12859-022-04869-7)
Supplement: Supplementary file 1 — Additional file 1. The official full name of 23 m6A regulators. [file 12859_2022_4869_MOESM1_ESM.docx]

| Official Symbol | Type | Official Full Name |
| --- | --- | --- |
| METTL3 | writers | Methyltransferase Like 3 |
| METTL14 | writers | Methyltransferase Like 14 |
| METTL16 | writers | Methyltransferase Like 16 |
| WTAP | writers | WT1 Associated Protein |
| VIRMA | writers | Vir Like M6A Methyltransferase Associated |
| ZC3H13 | writers | Zinc Finger CCCH-Type Containing 13 |
| RBM15 | writers | RNA Binding Motif Protein 15 |
| RBM15B | writers | RNA Binding Motif Protein 15B |
| YTHDC1 | readers | YTH Domain Containing 1 |
| YTHDC2 | readers | YTH Domain Containing 2 |
| YTHDF1 | readers | YTH N6-Methyladenosine RNA Binding Protein 1 |
| YTHDF2 | readers | YTH N6-Methyladenosine RNA Binding Protein 2 |
| YTHDF3 | readers | YTH N6-Methyladenosine RNA Binding Protein 3 |
| HNRNPC | readers | Heterogeneous Nuclear Ribonucleoprotein C |
| FMR1 | readers | FMRP Translational Regulator 1 |
| LRPPRC | readers | Leucine Rich Pentatricopeptide Repeat Containing |
| HNRNPA2B1 | readers | Heterogeneous Nuclear Ribonucleoprotein A2/B1 |
| IGF2BP1 | readers | Insulin Like Growth Factor 2 MRNA Binding Protein 1 |
| IGF2BP2 | readers | Insulin Like Growth Factor 2 MRNA Binding Protein 2 |
| IGF2BP3 | readers | Insulin Like Growth Factor 2 MRNA Binding Protein 3 |
| RBMX | readers | RNA Binding Motif Protein X-Linked |
| FTO | erasers | FTO Alpha-Ketoglutarate Dependent Dioxygenase |
| ALKBH5 | erasers | AlkB Homolog 5, RNA Demethylase |

Supplementary Table. The official full name of 23 m6A regulators.
